# Supplementary material for: Temperate Air Breathing Increases Cycling Performance in Hot and Humid Climate Environment
Source: Life (Basel). 2021 Sep 1;11(9):911. doi: 10.3390/life11090911 (PMC8465945; doi:10.3390/life11090911)
Supplement: Supplementary file 1 [file life-11-00911-s001.zip › life-1279077-supplementary.pdf]

Supplemental data of

Temperate Air Breathing Increases Cycling Performance in Hot and Humid Climate Environment

Clovis Chabert [1,†,*], Aurélie Collado [1,†] and Olivier Hue [1,*].

1. Laboratoire ACTES, Université des Antilles, UPRES-EA 3596 UFR-STAPS, Pointe-à-Pître 97110, Guadeloupe - France; aurelie.collado@univ-antilles.fr
† Both authors contributed equally to this work.
* Correspondence: olivier.hue@univ-antilles.fr (O.H.); clovis.chabert@inserm.fr (C.C.)

Psychological measurement

At the end of each session, subjects completed a short form of the Profile of Mood states (POMS-SF), whose results are presented in Supplemental Table S1. This questionnaire consists of 37 adjectives self-rated on a 5-point scale (from 0 to 4) and grouped into six mood factors: Tension–Anxiety, Anger–Hostility, Fatigue–Inertia, Vigor–Activity, Confusion–Bewilderment and Depression–Dejection.

Table S1. Scores associated to the Profile of Mood States questionnaires. Mood Disturbance = (Tension + Anger + Fatigue + Confusion + Depression) – Vigor. (n = 10; Mean±SEM). HA: Hot Air inlet (33 °C); TA: Temperate Air inlet (23 °C).

	HA			TA		
	**Mean**	**±**	**SEM**	**Mean**	**±**	**SEM**
Tension	5.0	±	2.0	3.5	±	1.7
Anger	1.9	±	0.8	3.1	±	1.4
Vigor	10.5	±	2.1	10.8	±	1.2
Fatigue	7.4	±	1.5	5.8	±	1.3
Confusion	2.9	±	0.9	2.5	±	1.3
Depression	1.0	±	0.3	1.4	±	0.7
Mood Disturbance	71.3	±	5.5	72.9	±	6.2

Indeed, none of the measured items presented in the Supplemental Table S2 is altered by the air condition. This result tend to be confirmed by the absence of effect observed on the Mood Disturbance index, which is calculated by subtracting Vigor score to (Tension + Anger + Fatigue + Confusion + Depression).

Visual analogic scales

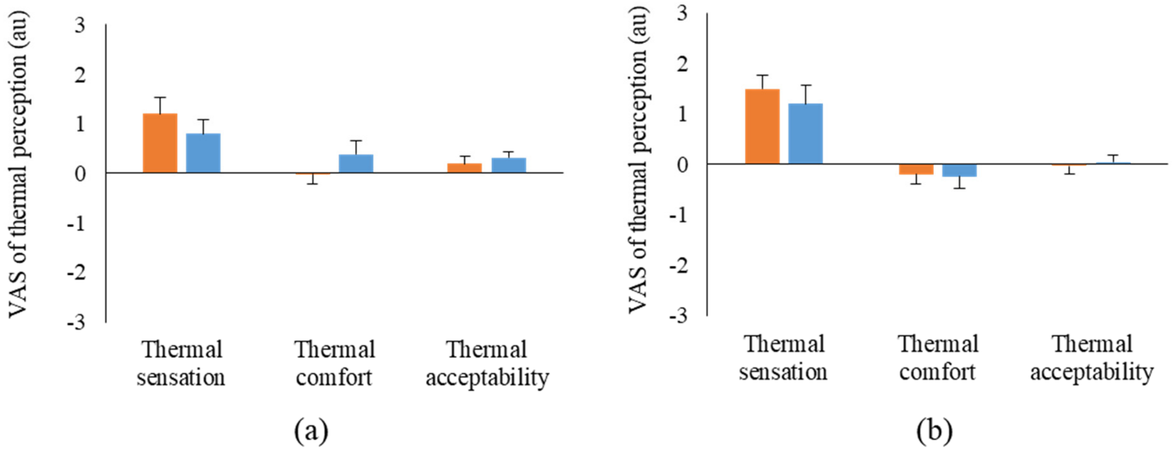

Figure S1. Graphical representation of the visual analogic scales focusing the thermal perception of subjects before start of cycling Pre-Fatigue (A) and Performance (B). (n = 9-10; Mean ± SEM). ■: Hot Air inlet (33 °C); ■: Temperate Air inlet (23 °C). V'E: V'E. VAS: Visual Analogic Scale.
